# Cultural adaptations made to existing implementation science theories, models, frameworks, or outcomes: a scoping review

**DOI:** 10.1093/tbm/ibag021

**Published:** 2026-04-24

**Authors:** Zoe Fehlberg, Samantha Croy, Madeline Graham, Marlena Klaic, Stephanie Best

**Affiliations:** Melbourne Implementation Research Group, School of Health Sciences, University of Melbourne, Melbourne, Victoria, Australia; Centre for Population Genomics, Murdoch Children’s Research Centre, Melbourne, Victoria, Australia; Centre for Population Genomics, Murdoch Children’s Research Centre, Melbourne, Victoria, Australia; Centre for Population Genomics, Garvan Institute of Medical Research, University of New South Wales, Sydney, New South Wales, Australia; Melbourne Implementation Research Group, School of Health Sciences, University of Melbourne, Melbourne, Victoria, Australia; Melbourne Implementation Research Group, School of Health Sciences, University of Melbourne, Melbourne, Victoria, Australia; The Royal Melbourne Hospital, Allied Health Department, Melbourne, Victoria, Australia; Melbourne Implementation Research Group, School of Health Sciences, University of Melbourne, Melbourne, Victoria, Australia

**Keywords:** implementation science, theories, models and frameworks, cultural adaptation, health equity, evidential injustice, scoping review

## Abstract

**Background:**

Cultural adaptation to implementation science research tools may be necessary to avoid biases in how evidence is collected and analyzed when working with multi-cultural communities.

**Purpose:**

This scoping review aimed to examine literature reporting community-specific adaptations to existing research tools associated with implementation science theories, models, frameworks, and outcomes.

**Methods:**

A pre-registered protocol informed by the PRISMA-SC guided the process. Six databases were searched from inception to April 2025. Articles were selected using a predetermined criterion. The purpose, type of, and adaptation process(es) were extracted, and a narrative synthesis informed by two adaptation frameworks was undertaken. Data on the application, testing, and impact of the adapted tool were extracted.

**Results:**

Twelve articles were included. Studies reported working with Indigenous, Black-American, Latinx, Hispanic, immigrant, or multi-ethnic communities. All adaptations were researcher-initiated, 92% were proactively planned, and 42% included community engagement. Half of the adaptations were to frameworks identifying factors influencing implementation. Adaptations largely involved changing the content, concepts, or the goals of the tool by integrating complementary cultural concepts or models. Six studies empirically and two hypothetically applied the tool. No study systematically evaluated the impact.

**Conclusions:**

Our scoping review found community-specific adaptations are occurring and provides insight into why and how. More research is needed into the impact of adaptation and whether it better equips those who use implementation research tools in practice with the ability to conduct research that provides policymakers and healthcare decision-makers with meaningful evidence to promote health equity.

## Graphical abstract

**Graphical Abstract:**
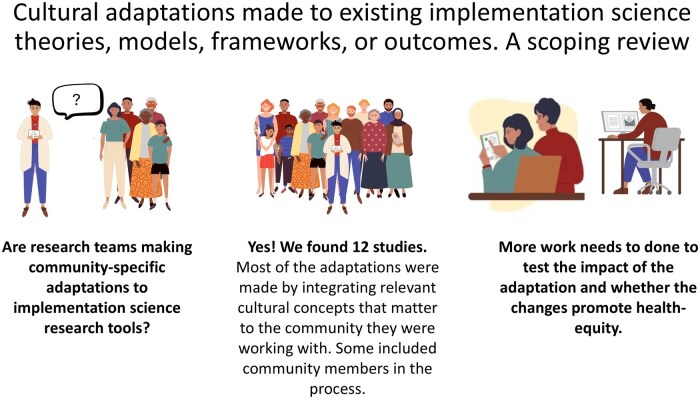

## Teaser

Our scoping review summarizes existing literature about how and why researchers are making adaptations to implementation science research tools when working with culturally diverse communities.

Implications **Research:** Researchers should prioritize systematic evaluation of the impact of cultural adaptations across a range of outcomes and how they may contribute to reducing biases in evidence and health inequities.

**Practitioners:** Those who use implementation science tools in practice may need to consider adapting tools and documenting the process when working with diverse communities.

**Policymakers:** Policymakers should recognize the importance of culturally adapted implementation science tools in generating more equitable and relevant evidence.

## Introduction

Implementation science is the “scientific study of methods to promote the systematic uptake of research findings and other evidence-based practices into routine practice” [1]. To achieve this aim, efforts are frequently structured using research tools, such as data collection instruments to inform, collect, and synthesize implementation evidence. These tools are typically associated with a theory, model, framework (TMF), or implementation outcome (e.g. acceptability). Engaging with relevant partners, including consumers and community, is fundamental to the discipline [2]. Depending on the context, this may mean working on projects with communities that have diverse cultural and ethnic identities. Implementation science is a relatively new scientific discipline with much of the foundational work conducted and continuing to be developed in Western regions, notably, North America, United Kingdom, and Australia [3], although this pattern is changing [4]. With a greater emphasis on community-defined evidence and collaboration with culturally diverse communities in implementation science projects [5, 6], researchers are questioning whether the available implementation science research tools make disciplinary assumptions based on Western notions that may unfairly fail to include differing worldviews and contexts [7–9]. The term for this phenomenon is evidential injustice [10], an important factor to consider in supporting principles of health equity in implementation science [11]. As the implementation science knowledge base rapidly advances and the demand for integrating its principles into research increases, it is timely to ensure that the approaches to generating evidence in the field, including data collection tools, are critically examined and revised to ensure they include the voices of the whole community. Otherwise, the field risks being ill-equipped to face implementation challenges in delivering equity-focused health care.

Equity-focused health care strives to overcome the unjust and avoidable differences in health outcomes and health care access between groups characterized by structural, interpersonal, institutional, and social factors, including socioeconomic status, gender, geographic location, ethnicity, and migration status, to name a few [12]. Ethnicity-related health equity is a concept that centers the strengths and experiences of individuals based on a shared geographic origin and ancestry, alongside a shared history, language, and set of beliefs and customs [13]. Reducing health inequities, including those related to health and ethnicity, is an international goal [14] with many high-income countries prioritizing health equity-focused research and policy [15, 16]. Therefore, health equity is a focus of implementation science and has drawn attention to how TMFs can include aspects of health equity [5]. In a scoping review, Gustafson *et al.* identified and described 15 equity-focused TMFs that have been used to support implementation activities in populations who experience ethnicity-based health inequities [17]. Nine of the TMFs were newly developed (e.g [18].), three were amended (e.g [5].), and three had been operationalized in an equity context (e.g [19].). The majority of the TMFs were characterized with the explicit aim of reducing inequities and were designed to be applicable across various contexts and populations.

Cultural adaptation provides an opportunity for implementation science to facilitate health equity efforts by improving the reach of health interventions to all groups of people who stand to benefit [20]. Cultural adaptation involves improving the compatibility of an intervention through careful consideration and incorporation of the intended recipients cultural values, norms, customs, belief systems, and ways of making meaning [21]. This idea has seen numerous cultural adaptations to interventions in a variety of healthcare settings, including diabetes management and mental health [22, 23]. However, this work has not yet extended to determining whether cultural adaptation to the research tools used in implementation science is needed to minimize the risk of evidential injustice [20], and how best to undertake the process. Cultural adaptation of health measures in other fields has produced guidelines for the process of translation and cross-cultural adaptation of self-reported quantitative health questionnaires, e.g [24, 25], or adaptations related to concepts of health and wellness [26]. However, whether these approaches are used or how they may apply to implementation science is not well known.

To address the described gap in the literature, this scoping review aims to examine literature reporting community-specific adaptations to implementation science research tools. The objectives of the review were to

- Identify existing TMFs or outcomes that have been culturally adapted when working with culturally and ethnically diverse communities in high income country health settings.
- Summarize the types of adaptations, reasons, and processes taken for making them.
- Examine how the adapted research tools have been applied, tested, and the impact of the adaptation.

## Methods

### Rationale, design, and protocol registration

We conducted a scoping review as the aim was broad and exploratory, rather than to compare or evaluate an approach or intervention [27]. The PRISMA Extension for Scoping Reviews (PRISMA-ScR) was used to guide the process and reporting [28], and a review protocol was registered with Open Science Frameworks (ID: osf.io/srymk).

### Author team

We wish to acknowledge how our perspectives and approach to the review are shaped by our positionality and have included a statement as Supplementary Material 1.

### Search strategy and eligibility criteria

The search strategy was built in consultation with an experienced health services librarian following the Peer Review of Electronic Search Strategies (PRESS) checklist [29]. The search terms for implementation TMFs or outcomes were developed from an online repository of available frameworks [30] and additional TMFs specific to health equity [31]. See Supplementary Material 2 for an example of the search strategy. In addition, the reference lists of included studies were searched for possible articles. The eligibility criteria were structured using the Population, Concept, and Context (PCC) mnemonic to reflect the focus on research tools as a concept and are presented in Table 1.

**Table 1:** Eligibility criteria (inclusion/exclusion) structured using the PCC (Population, Concept, and Context) mnemonic.

	Inclusion criteria	Exclusion criteria
**Population**	Culturally and ethnically diverse communities or people who work with these communities (e.g. researchers, program developers, policy makers).	Articles reporting on the implementation of a health program aimed at the general population or where the description of the intended population is unclear.
**Concept**	Articles describing a cultural adaptation to an existing implementation science theory, model, framework, or disciplinary outcome.	Articles reporting the development of a new theory, model, framework, or disciplinary outcome without an adaptation component.
**Context**	Intended application/use within high-income country health settings.	Intended application/use outside of health, e.g. education, justice, social system.
**Source type**	Peer-reviewed articles including original empirical research of any study design, written in English.	Reviews, commentaries, debates, protocols, editorials, theses, book chapters, grey literature, or conference proceedings. Non-English publications.

### Information sources

We searched six databases (Medline, Global Health, PsycINFO, CINAHL, Scopus, and Web of Science) spanning a range of health fields from inception until 16 April 2025. No limits were used. Corresponding authors were contacted for further information if considered necessary.

### Study selection

All sources were downloaded and imported into the Covidence platform [32], where duplicates were automatically and manually removed. Title and abstracts of each source were screened in Covidence independently by Z.F. and M.G. using a double-blinded process, against the inclusion/exclusion criteria. Full texts were screened using the same process. Disagreements were first discussed between the pair of screeners before being taken to the authorship group, if unresolved.

### Data extraction

Data were extracted from eligible studies and recorded in a purpose-created spreadsheet that was tested and revised by the authorship team. One researcher (Z.F.) extracted all data. A second researcher (M.G.) initially reviewed the extracted data for a third of the studies to identify errors or missing information. With no changes made, the data validation and review process was considered complete. Two adaptation frameworks, the Framework for Reporting Adaptations and Modifications to Evidence-based Interventions (FRAME) [33] and Ecological Validity Model (EVM) [34], were used to categorize the process(es) undertaken for the cultural adaptation. The FRAME is an implementation science framework established to record adaptations to an intervention. The EVM characterizes eight adaptation dimensions. Despite their intention for intervention adaptation, each framework was amenable to the context of the review. See Supplementary Material 3 for the FRAME and EVM data extraction template, including descriptions.

### Data analysis and synthesis

Frequency counts were used to analyze and present categorical data. A narrative synthesis [35] approach was used to describe the cultural adaptation process(es) undertaken using the FRAME components and EVM dimensions, and if and how the tool had been applied and tested.

## Results

### Study selection

The search returned a total of 6361 records. After the removal of duplicates, 2784 remained for title and abstract screening. Following title and abstract screening, 61 eligible full-text articles were assessed, with 12 meeting the inclusion criteria for review [36–47] (see Supplementary Material 4). No additional articles were found by searching the full-text articles’ reference lists. Interrater reliability varied (screening: 3.9% disagreement and full text: 32%). The increase in conflicts during full-text assessment largely arose because of the challenges in the nuanced difference between adaptations made for cultural reasons and adaptations made to improve the research process. These discrepancies were resolved between the reviewer pair by further clarifying what cultural adaptation is.

### Study characteristics

The characteristics of the 12 articles are provided in Table 2. In summary, all the studies were recently published 2019–2025, and most were from North America (USA, *n *= 5 and Canada, *n *= 4) [36, 37, 40, 41, 43, 45–47]. One study was set across New Zealand and Canada [39], and the remaining two studies were from New Zealand [38] and Australia [44]. Five studies reported adaptations when working with Indigenous peoples [36, 38, 39, 43, 44] and five included Black-American, Latinx, Hispanic, immigrant, or multi-ethnic communities [40, 41, 45–47]. Two studies generated by the same initiative took an intersectional approach, with culture as one factor [37, 42]. Qualitative methodology was the most frequently used study design (*n *= 4) [36, 41, 43, 44], followed by multi [37, 38, 42] or mixed methods [39, 40, 47] (*n *= 3, each), and two case study designs [45, 46]. Half of the studies reported adaptations to the CFIR, which is a determinants framework (i.e. used to examine and explain factors that influence implementation) [36, 40–42, 44, 45]. Another determinants framework (Promoting Action on Research Implementation in Health Services, i-PARIHS) was adapted in one study [47]. A behavioral framework (i.e. used to characterize factors that influence behavior), the Theoretical Domains Framework (TDF) was adapted twice [37, 43]. Two studies adapted process models (i.e. structured approaches to guide the process of implementation), the (Equity-based Framework for Implementation Research [EquIR], and Exploration, Preparation, Implementation, Sustainment [EPIS]) [38, 46] and one adapted an assessment tool (Readiness Assessment for Pragmatic Trials [RAPT] model) [39]. Table 2 also includes a summary of the adaptation, of which, seven integrated a culturally relevant theory or element (e.g. CFIR + fundamental cause theory) into the existing tool [36, 37, 40, 43–45], four integrated a cultural component (e.g. the EquIR + the cultural context) to make a new framework [38, 39, 46, 47], and one combined photovoice as a culturally sensitive research method with an implementation framework [41]. Eight of the adapted tools were applied either empirically (*n *= 6/8) [36, 40, 41, 43, 44, 47] or hypothetically (*n *= 2/8) [37, 45], however, none underwent further testing or systematic assessment to determine the impact of the adaptation. Both topics are discussed in a later section.

**Table 2:** Characteristics of the included studies.

Study Year	Country	Intended community/population	Study design	Aim of adaptation	IS tool	Tool category	Adaptation summary	Was the tool applied	Was further testing reported
Barker 2025	Canada	First Nations People	Qualitative	Secondary	CFIR	Determinants framework	CFIR + First Nations Perceptions of Health and Wellness model	Yes	No
Etherington 2020	Canada	Diverse, population not specified	Multi-methods	Primary	TDF	Behavioral framework	TDF + Intersectionality lens	Yes	No
Gustafson 2024	New Zealand	Māori and Pacific peoples	Multi-methods	Primary	EquIR	Process model	EquIR + cultural context = new model	No	No
Hikaka 2024	New Zealand and the USA	Māori, American Indian, Native Alaskan, Kanaka, Maoli, Native Hawaiian peoples	Mixed methods	Primary	RAPT model	Assessment tool	RAPT + equity considerations for developing trials with Indigenous Peoples = RAPT-I	No	No
Jacobs 2023	USA	Black, Latino, Hispanic, and African immigrant populations	Mixed methods evaluation	Primary	CFIR	Determinants framework	CFIR + Culturally Responsive Evaluation Framework	Yes	No
Johnson 2024	USA	Hispanic and non-Hispanic men	Qualitative	Primary	CFIR	Determinants Framework	CFIR-based focus groups + photovoice	Yes	No
Robak 2024	Canada	First Nations people	Qualitative	Secondary	TDF	Behavioral framework	TDF + cultural constructs	Yes	No
Rodrigues 2023	Canada	Diverse, population not specified	Multi-methods	Primary	CFIR	Determinants framework	CFIR + Intersectionality lens	No	No
Sebastian 2021	Australia	Australian Indigenous people	Qualitative	Secondary	CFIR	Determinants framework	CFIR + cultural suitability construct	Yes	No
Senier 2019	USA	Multi-ethnic communities	Case study	Primary	CFIR	Determinants Framework	CFIR + fundamental cause theory	Yes	No
Thompson 2022	USA	Black and Latina transgender women	Case study	Primary	EPIS	Process model	EPIS + trans health context = new model	No	No
Woodward 2019	USA	Black people	Mixed methods	Primary	i-PARIHS	Determinants Framework	i-PARIHS + the Health Care Disparities Framework = Health Equity Implementation Framework	No	No

CFIR, Consolidated Framework for Implementation Research; EquIR, Equity-based Framework for Implementation Research; EPIS Framework, Exploratory, Preparation, Implementation, Sustainment; i-PARIHS, integrated-Promoting Action on Research Implementation in Health Services; IS, implementation science; RAPT Model, Readiness Assessment for Pragmatic Trials Model; TDF, Theoretical Domain Framework.

### Adaptation processes

#### Framework for Reporting Adaptations and Modifications to Evidence-based Interventions (FRAME)

As summarized in Table 3, when synthesized according to the FRAME components, all but one [44] of the adaptations were made proactively (i.e. deliberately planned) and prior to data collection (*n *= 11). All adaptations were research team-initiated and were described (*n *= 7/12). Teams either included individuals from the community intended to benefit from the research (*n *= 4/7) [36, 39, 43, 46] or experts from relevant areas (*n *= 3/7) [37, 38, 42]. “Improving the effectiveness” of the existing tool (e.g. increase the ability of the tool to capture relevant data) was the most frequent goal for the adaptation (*n *= 9) [37, 39, 40, 42–47],. The goal of the remaining three studies was “improving the fit with recipients” by adapting the interview schedule for the population [36], “improving the feasibility” of conducting equity-focused research by developing a contextualized process model [38], and “increasing reach or engagement” by adapting the methods to attract participants [41]. All the adaptations were made to both support researchers or practitioners in practice and for the benefit of the intended recipients. That is, the adaptation was made for “individuals who share a particular characteristic”. Half of the studies could be characterized as a singular modification (e.g. tailoring the tool) [36, 40, 41, 43, 44, 46], the other half were characterized by two or three modifications (e.g. tailoring the tool and changing the materials by providing a user guide for the adapted version) [37–39, 42, 45, 47]. When reported (*n *= 9/12), the people involved in the adaptation process were either both researchers and individuals from the intended communities (*n *= 4/9) [36, 38, 43, 46], or researchers only (*n *= 5/9) [37, 39, 41, 42, 47]. The number of contributors included was poorly reported, as were the recruitment methods.

**Table 3:** Details of the cultural adaptations made categorized according to the Framework for Reporting the Adaptation and Modification to Evidence-based Interventions (FRAME) [33] and Ecological Validity Model (EVM) [34].

Study year	**Proactive or reactive adaptation** (FRAME)	**Who** participated in the decision to adapt the tool (FRAME)	**Timing** in relation to data collection (FRAME)	**Goal and Reason** for adaptation (FRAME)	**For whom** was the adaptation made (FRAME)	**Nature** of the adaptation (FRAME)	**Who** participated in the adaptation process and recruitment strategy (FRAME)	Methods	**What** was adapted (EVM)
Barker 2025	**Proactive**	**Research team** Members of a First Nations Health Authority and Indigenous peer researchers	**Prior**	**Improve fit with recipients** Ensure interview schedule was accessible, culturally safe and produced meaningful data	**Researchers who work with Indigenous people** **Indigenous People**	**Tailoring** CFIR domains/constructs based on the First Nations Perceptions of Health & Wellness model	People with lived experience and research team (*n* = NR) Recruitment, NR	1. Feedback from research team and people with lived experience 2. Analyzed CFIR-coded data according to cultural model	**Concepts** interview schedule and analysis of CFIR domains were related to the rings of the cultural model
Etherington 2020	**Proactive**	**Research team** implementation science practitioners/researchers/trainees, intersectionality experts, experts in community health, kinesiology, medicine, physical therapy, psychology, and sociology	**Prior**	**Improve effectiveness** Intersectionality offers a nuanced and complete account of context and factors that intersect to shape individual decision-making and behavior	**Implementation researchers/practitioners**	**Tailoring** TDF constructs for intersectionality **Changes in materials** development of intersectionality prompts and user guide	Framework committee (*n *= 17) Sub-committee (*n* = NR) Recruitment, NR	1. Brainstormed how to incorporate intersectionality into domains 2. Developed and revised overarching considerations, prompts and user guide	**Goals** provide researchers with overarching considerations and interview prompts to incorporate intersectionality into barrier and enablers research
Gustafson 2024	**Proactive**	**Research team** Expertise in health equity, Māori health and the local health system	**Prior**	**Improve feasibility** A context-appropriate process framework was needed	**Researchers** **Non-Indigenous health care providers**	**Adding** key cultural/contextual elements **Tailoring** process steps for the context and expected users (non-Indigenous health providers)	Interviews with lead researchers (*n *= 12) and health service leaders (*n *= 13) Workshop with research team (*n *= 7) Advisory groups (*n* = NR) Recruitment, based on expertise	1. Literature review to identify TMFs 2. Interviews with contributors to identify key elements 3. Workshop to select TMF, modify steps, match against interview findings 4. Iterative revisions in consultation with Māori and community advisory groups	**Content and concepts** principles of the Treaty of Waitangi and whānau-extended family) **Goals** equity-focused implementation **Persons** suitable for health care providers
Hikaka 2024	**Proactive**	**Research team,** including an Indigenous health services researcher	**Prior**	**Improve effectiveness** The original RAPT domains defined by discussion amongst experts do not explicitly consider equity	**Researchers conducting trials with Indigenous communities**	**Expanding** of domains to incorporate important Indigenous considerations **Adding** domains to support Indigenous communities’ power within research	Survey (*n *= 21) and interviews (*n *= 7) with self-identified researchers in clinical setting and Indigenous research Recruitment, authors identified from a literature review, snowball, directors of relevant clinical/Indigenous research centers and networks	1. Survey to rate domain appropriateness and need for adaptation 2. Draft version RAPT-I 3. Interviews with respondents to further explore appropriateness and revisions 4. Revised version RAPT-I sent to survey participants for feedback	**Goals** assess trial cultural appropriateness and readiness **Content** to include Indigenous Sovereignty, Acceptability to Indigenous Communities, Risk of Research, Research Team Experience, Established Partnership
Jacobs 2023	**Proactive**	**Research Team** NR	**Prior**	**Improve effectiveness** Develop evaluation questions and data collection instruments that capture cultural considerations and implementation evidence	**Researchers**	**Changes in materials** developed CFIR-informed evaluation questions and instruments	NR	NR	**Content** evaluation questions and data collection instruments structured by the CFIR
Johnson 2024	**Proactive**	**Research team** NR	**Prior**	**Increase reach, engagement** CFIR reliance on data collection methods (interviews and focus groups) may limit community engagement	**Target intervention group**	**Changes in materials** to create a CFIR-informed photovoice study	Research team (*n* = NR) Implementation Science Coordination Initiative (*n* = NR) Recruitment, NR	NR	**Methods** a CFIR-informed photovoice approach
Robak 2024	**Proactive**	**Research team** Settler allies and Indigenous researchers	**Prior**	**Improve effectiveness** The TDF domains “knowledge” and “Environmental Context & Resources” did not support Indigenous perspectives and are based on Euro-Western and biological standpoints	**Researchers**	**Tailoring** TDF constructs related to “culture” and “context” to understand and support Indigenous perspectives	Researchers (*n* = NR) Community leaders and end users (*n* = NR) Recruitment, NR	1. Adaptations to interview schedule and coding guide (not provided) 2. After analysis community (leaders and end users) workshop reinforced the adaptations	**Concepts** of cultural awareness sensitivities and colonialism and intergovernmental boundaries, legislation, political and economic jurisdictions
Rodrigues 2023	**Proactive**	**Research team** implementation developers, implementation science trainees, TMFs experts, individuals with training in intersectionality, critical feminist scholar	**Prior**	**Improve effectiveness** Currently, there are few TMFs to guide implementation science researchers to use intersectional considerations	**Implementation researchers/practitioners**	**Tailoring** CFIR constructs for intersectionality **Adding** constructs related to intersectionality **Changes in materials** development of intersectionality prompts and user guide	Framework committee (*n *= 17) Sub-committee (*n *= 7) Recruitment, NR	1. Prioritized CFIR constructs for adaptation 2. Brainstormed how to incorporate intersectionality into constructs 3. Developed and revised framework, prompts and user guide	**Content and Concepts** considerations and prompts to help researchers reflect on how individual identities and structures of power may play a role in implementing evidence-based interventions
Sebastian 2021	**Reactive**	**Research team** NR	**After**	**Improve effectiveness** None of the CFIR constructs sufficiently included the cultural suitability of an intervention	**Researchers**	**Adding** “cultural suitability” as a construct during analysis	NR	NR	**Content and concepts** related to elements of the program that acknowledge Indigenous ways of living, reflect experiences, environment and world view
Senier 2019	**Proactive**	**Research team** NR	**Prior**	**Improve effectiveness** Few frameworks examine how implementation affects health inequities and account for the broader social, political and economic forces that hinder access to care	**Researchers**	**Reorder** to have a nested model with patients, providers and families at the center, surrounded by fundamental causes of disease, implementation strategies, and relevant contributors to involve **Expand** the CFIR “outer setting” to include a broader array of forces affecting health care delivery	NR	NR	**Goals** integrate sociology theory into implementation science and improve the ability to understand contextual factors and involve relevant contributors
Thompson 2022	**Proactive**	**Research team** Academic researchers (cisgender), Steering committee members (transgender), and community members (transgender and/or people of colour)	**Prior**	**Improve effectiveness** To identify unique barriers and facilitators to trans health intervention implementation	**Researchers working in trans health implementation**	**Tailoring** EPIS constructs for trans health equity-focused implementation	Research team (*n* = NR) 2 Steering committees (*n *= 10 per) Community members (*n *= 40+) Recruitment, NR	1. Transgender community engagement (World Café meetings) 2. Leveraged steering committees to analyze findings and adapt the EPIS to the context and apply to as case studies	**Goals** guidance for trans health implementation
Woodward 2019	**Proactive**	**Research team** The lead investigator is a white female researcher	**Prior**	**Improve effectiveness** A framework for healthcare disparity implementation challenges is needed to understand implementation problems and select implementation strategies	**Implementation researchers/practitioners**	**Reordering** to place the clinical encounter at the center of framework **Extending** framework determinants to include equity considerations	Research team (*n* = NR) Recruitment, NR	NR	**Content and Concepts** To be better equipped to understand how implementation science can address social disparities

EPIS Framework, Exploratory, Preparation, Implementation, Sustainment; CFIR, Consolidated Framework for Implementation Research; EquIR, Equity-based Framework for Implementation Research; i-PARIHS, integrated-Promoting Action on Research Implementation in Health Services; NR, not reported; RAPT-I Model, Readiness Assessment for Pragmatic Trials Model -Indigenous; TDF, Theoretical Domain Framework; TMFs, Theories, Models, Frameworks.

#### Ecological Validity Model

**Figure 1:**
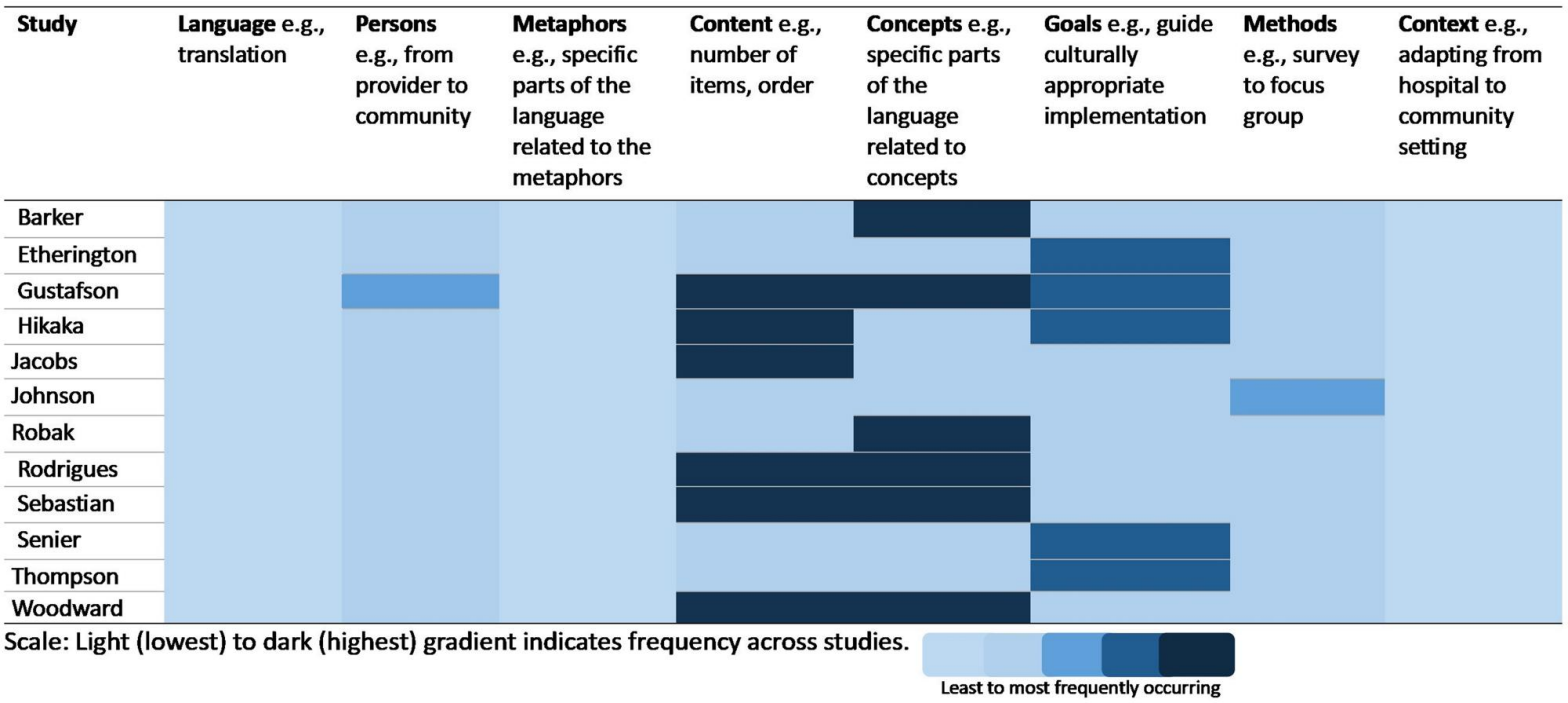
Heatmap of the frequency of Ecological Validity Model components by study and across studies.

When synthesized according to the EVM dimensions (Figure 1) and described in Table 3, seven studies could be characterized as adapting one EVM dimension [36, 37, 40, 41, 43, 45, 46]. The other studies are characterized by two [39, 43, 44, 47], or four [38] adaptation dimensions. Across the studies, adaptation to the “concepts” (e.g. specific parts of the language related to concepts) and/or the “content” of the tool (e.g. the number of items or their order) were the most frequent type of adaptation [36, 38–40, 42–44, 47], followed by altering the “goal” of the tool (e.g. from guiding implementation to guiding culturally appropriate implementation) [37–39, 45, 46]. Cultural adaptations relating to “persons” (e.g. from provider to community) [38] or “methods” (e.g. survey to focus group) [41] were identified in two studies. None of the studies adapted the “language” (e.g. translation) or “context” (e.g. adapting from hospital to community setting).

#### Methods

For the seven studies that provided details of the methods used to inform the cultural adaptation (Table 3) [36–39, 42, 43, 46] five were made over iterative refinement using a range of methods to either understand what parts or aspects of the tool to adapt [37, 39, 42], or gather contributors’ opinions on important cultural considerations to incorporate [38, 46]. All were then followed by a series of opportunities for continued feedback and refinement with different contributors. The other two studies made the adaptations at a singular point in time, with community involvement [36, 43]. No study reported using a theory to inform their methods.

### Application of the adapted tool

The culturally adapted tools were applied in eight of the studies. Empirical applications included semi-structured interviews (*n *= 4/8) [36, 43, 44, 47], a photovoice study [41], and a program evaluation [40]. Two studies hypothetically applied the adapted tool to case examples [37, 45]. Participants were either the planned recipients of an intervention (*n *= 3/8) [41, 45, 47], health care professionals or program staff (*n *= 2/8) [37, 44], or both (*n *= 3/8) [36, 40, 43].

### Further testing and assessment of the impact of the cultural adaptation

No study reported further testing of the adapted tool. Only one of the included studies stated that they assessed the impact of the adaptation using a predefined measure (feasibility); however, the methods used, or results were not reported [47]. One study asked respondents involved in the adaptation process whether they would use the adapted tool and reported that 72% selected yes [39]. More often, when the tool had been applied in the study, the discussion included a brief description of the authors’ perceived benefits and pitfalls of the adapted tool. A summary of the application and impact is provided as a table in Supplementary Material 5.

## Discussion

As implementation science increasingly becomes embedded in health research, it is timely to recognize whose voices are being captured by the available research tools and, where necessary, revise these tools. By doing so, the evidence that is used to inform the implementation of health care may be more inclusive, at less risk of biases and hold greater potential for equitable impact [17, 24]. This scoping review progresses knowledge of how cultural adaptation is being used to enhance implementation science by examining evidence of community-specific adaptations made to existing research tools. The findings from the review provide a starting place to progress the greater inclusion of diverse perspectives in how data are collected and analyzed, contributing to evidential justice.

The 12 included peer-reviewed studies reported adaptations to six different implementation science research tools. Although the studies were conducted in a handful of countries, the intended beneficiaries of the adaptations varied, including projects working with Indigenous, Black-American, Latinx, Hispanic, immigrant, or multi-ethnic communities. All studies were published in recent years (2019–2025), and could be characterized as researcher-initiated, albeit some of the research teams included community members. These findings indicate the growing attention and research community’s willingness to undertake health equity-focused research [48].

### Adaptation and generalizability of TMFs

Implementation science TMFs are developed with generalizability as a central tenet [49]. That is, as demonstrated with the CFIR, they can be applied and yield valuable findings in disparate contexts and settings [50]. However, the generalizability of a TMF may come at the cost of reduced sensitivity to contextual factors vital to understanding the causes and drivers of health equity. This consequence was noted in some of the included studies as a reason for adapting the tool, e.g [38]., as were the limitations of frameworks based on Euro-Western epistemological and biological standpoints [43] or the lack of diversity with which the initial tool was developed [39].

Examples of the adaptations illustrated in this review may provide some ability to strike a balance between generalizability and context. For example, Etherington *et al.* [37, 42] supplemented the CFIR and TDF with an intersectionality lens that may enable researchers to collect and synthesize culturally sensitive evidence without being bound to a specific context. Born from Black feminist activism and gender scholarship, an intersectional approach may provide a useful theoretical orientation to examine cultural-inequities as it recognizes that social identities are multifaceted, shift among contexts, and intersect with systems of power and oppression [51]. When used in practice, Fontaine et al. found the intersectionality-adapted TDF provided a nuanced understanding of barriers and enablers and equity-focused implementation strategies to assist the delivery of hepatitis C point-of-care testing [52].

### Integrating interdisciplinary epistemology

Coalescing academic fields and epistemologies is not new to implementation science. Implementation science is inherently multidisciplinary, drawing from diverse fields including behavioral science, health equity research, organizational psychology, and public policy and health disparities research. Melding implementation science with a model or theory from another discipline was a feature of many of our included studies. One example is Barker *et al.* [36], who overlaid the CFIR with a local First Nations Perceptions of Health and Wellness Model. The addition facilitated the refinement of a culturally appropriate interview guide and supported contextualized analysis of data. Also starting with the CFIR, Senier *et al.* [45] integrated the framework with fundamental cause theory to allow for greater consideration of how broader social, political, and economic forces affect implementation in a way that leads to or entrenches health inequities. Going forward, it will be important to better understand ways to identify beneficial epistemologies to integrate [9], determine the impact of the decision on the original tool, and assess the added value.

### Supporting proactive and reactive adaptation

All but one of the included studies made proactive adaptations to the research tools before data collection. The other study reported what could be considered an “on-the-fly” adaptation [44]. It is likely that impromptu and reactive, though underreported, adaptations are being made and form an important part of practice. Regardless of whether the adaptation process is intentional and made from the outset or in response to what is unfolding, deft adaptation relies on the ability of those involved to first determine the limitations of the tool and level of revision required. Involving community members as part of the research team could be key, as seen in over half of the studies including communities. However, there were inconsistencies in the reporting of the methods used, who and how community individuals or groups involved in the adaptation process were selected, and the research team members’ positionality (i.e. how their worldview may be shaped by their social and political standing) [53]. These omissions make it challenging to know how to support both proactive and reactive approaches to adaptation, including the ability to know when, to what extent, and how to go about adaptation.

### Gaps identified and future research directions

Our review identified a significant gap; no studies adapted tools associated with the eight established implementation outcomes [54], such as “acceptability” or related measurement tools e.g. “Acceptability Intervention Measure” [55]. One of the included studies [17] adapted the (EquIR) framework, which stipulates “measuring and monitoring” implementation outcomes. The authors provided equity considerations for each outcome; however, these were not accompanied by real-world applications or examples. These prompts provide those planning projects with guidance, yet how equity considerations are embedded in the measurement of implementation science outcomes (e.g. does feasibility mean different things to groups?) and the impact on the quality of the evidence generated and implications for implementation and health, remains uninvestigated. Given how valuable these outcomes are to supporting context-driven implementation efforts [54], future work to determine whether the available tools associated with the outcomes are culturally appropriate is needed.

If we look beyond the context of this review, implementation outcome research tools are being translated into different languages. For example, research tools associated with acceptability, appropriateness, and feasibility [67] have been cross-culturally adapted into Spanish [56], and Malay [57]. Cross-cultural adaptation involves consideration of language and cultural idioms, a process informed by established guidelines, requiring multiple steps, including further psychometric testing, taking substantial time and resources to develop [24]. Our search did not find any examples of cross-cultural adaptation which may be relevant in communities that speak languages other than English, such as migrant populations. Assumed and required proficiency in English is a known barrier to equitable and inclusive research [58]. Determining whether the intensive process of cross-cultural adaptation, as recommended from other health areas, e.g. [59], is required for meaningful implementation research with culturally and linguistically diverse communities is warranted.

Evaluating the impact of the adaptation on the existing tool is critical to understanding the added value. Some of the included studies applied the adapted tool in practice, to case examples or have since been applied. For example, The Health Equity Implementation Framework has been applied in various settings [60, 61]. However, none of the included studies investigated the potential impact of the adaptation against a predefined outcome or undertook further testing. For quantitative measures, this might include testing psychometric properties, e.g. reliability, validity, and responsiveness etc. [62]. Or, as discussed above, revalidation, or cross-cultural validation. For adapted tools or methods aligned more to qualitative research paradigms or the practical process of implementation, other outcomes to consider could include power dynamics or fidelity to methodology [63]. Practical considerations such as feasibility and usability of adapted tools should also be evaluated and present a measurement issue for the field [64]. These limitations, and other gaps we have identified, signpost to areas of future research being,

- How to determine what type of and level of adaptation is required for meaningful implementation research.
- Whether research tools associated with implementation science outcomes, e.g. acceptability and feasibility require adaptation.
- Investigate through further testing the impact of the adaptation by comparing the effectiveness of original versus adapted tools or against appropriate outcomes, including psychometric properties, usability, outcomes related to the quality of the evidence generated, and implications for implementation and health equity.

### Limitations

A potential limitation of the review is our use of broad search terms for the population, such as “people of color” rather than an exhaustive list of specific communities. We searched the peer-reviewed academic literature, enquiry into the grey literature could further knowledge on the topic. The review only included studies that signified an adaptation in their abstract, excluding possible studies where the adaptation is first mentioned in the main article. We limited our search to high-income country health settings. Assessment of the adaptation impact was limited to noting the judgements of the authors, and therefore, no further analysis was conducted.

### Conclusions

Our scoping review found that community-specific adaptations are increasingly being made to implementation science research tools. The studies identified in the review were synthesized using two frameworks to support applicable knowledge of how and why the adaptations occurred. We also found gaps in understanding the impact of cultural adaptation and whether they contribute to inclusive research or may result in unintended consequences. The intention of the review is to bring attention to efforts to promote evidential justice and equip those who use implementation research tools in practice to better conduct implementation research. Doing so may provide policymakers and health care decision-makers with evidence that has a meaningful impact and promotes health equity.

## Supplementary Material

ibag021_Supplementary_Data

## Data Availability

No data were generated with this study.

## Welfare of animals

This article does not contain any studies with animals performed by any of the authors.

## Artificial intelligence

Artificial intelligence was not used in the development of this manuscript.

## Transparency statements

Study registration: This study was registered with Open Science Frameworks (ID: osf.io/srymk). Analytic plan pre-registration: The analysis plan was formally pre-registered with Open Science Frameworks (ID: osf.io/srymk). Analytic code availability: All materials used to conduct the study are presented in the article.
