# Supplementary material for: Cultural adaptations made to existing implementation science theories, models, frameworks, or outcomes: a scoping review
Source: Transl Behav Med. 2026 Apr 24;16(1):ibag021. doi: 10.1093/tbm/ibag021 (PMC13108720; doi:10.1093/tbm/ibag021)
Supplement: ibag021_Supplementary_Data [file ibag021_supplementary_data.docx]

**Supplementary Information**

Supplementation Material 1. Positionality Statements

The review team consists of experts in implementation science and applied social research, employed at academic institutions and medical research institutes. No external experts were invited to review the findings and our interpretations.

ZF: I am a White, Australian-born settler, descendant of European immigrants, who lives on the unceded lands of the Wurundjeri Woi-wurrung people of the Kulin Nation. I have a background in public health and currently work and am undertaking a PhD in implementation science at an academic institution. I understand that the disciplines in which I have trained, and work are, and continue to be impacted by colonisation. I acknowledge the systems and structures which afford me unjust privilege. As I continue my learning through my PhD, I am committed to improving my understanding and practice around anti-colonial research and to be guided by people whose experiences are different than my own.

SC: I am a Southeast Asian migrant to Australia of mixed Asian and European heritage. I live and work on the unceded lands of the Wurundjeri Woi Wurrung people of the Kulin nation in Naarm. I was trained in anthropology and sociology in both Singapore and Australia and acknowledge the historical role of both these disciplines in colonial projects. I am committed to the methodological movements in these fields that aim to dismantle structures of oppression.

MK: I am a cis-gendered white woman of European heritage from a non-English speaking family, born in Whyalla on the traditional Country of the Barngarla people. Growing up in a migrant family in Australia has shaped my awareness of privilege and inequities, which informs my commitment to equity-focused implementation science. I now live and work on the unceded lands of the Wurundjeri people of the Kulin Nation.

MG: I am a white cisgender Australian woman with an educational and professional background in digital tools and public health. I am PhD student researching public health and implementation science and received my education and live on the lands of the Wurundjeri Woi-wurrung and Bunurong peoples of the East Kulin Nations.

SB: I am a white cis-gender dual national (UK/Australia) woman. I received my tertiary education in England and Wales. My work and life experiences, initially as a chartered physiotherapist and now full time academic, have been international, including the Wales, England, Singapore, Malawi and currently Australia. I now live and work on Wurundjeri lands.

Supplementary Material 2: Example of the OVID – Medline search strategy. Note, no limits were used for any database.

Supplementary Material 2: Example of the OVID – Medline search strategy. Note, no limits were used for any database.

OVID - Medline

| **#** | **Query** |
| --- | --- |
| 1 | implementation science.tw,kf. or exp Implementation Science/ |
| 2 | ("implementation research" or "dissemination research" or "dissemination and implementation" or "knowledge translation" or "operations research").tw,kf. |
| 3 | (implementation adj3 (framework* or theor* or model* or outcome* or instrument* or measure*)).tw,kf. |
| 4 | ("Proctor* outcome*" or "Proctor* taxonomy" or "Consolidated Framework for Implementation Research" or "Theoretical Domains Framework" or "Behavio?r Change Wheel" or COM-B or "Determinants of Implementation Behavio?r Questionnaire" or "Theoretical Framework of Acceptability" or "Reach Effectiveness Adoption Implementation Maintenance" or RE-AIM or "The Knowledge-to-Action Framework" or "Diffusion* of Innovation*" or "theory of change" or "Acceptability Intervention Measure" or "Intervention Appropriateness Measure" or "Feasibility Intervention Measure" or "ORgani#ational Readiness for Implementing Change" or "readiness tool" or "Normali#ation Process Theory" or "Expert Recommendations for Implementing Change" or ERIC or "Dynamic Adaptation Process" or "Exploration, Adoption/Preparation, Implementation, Sustainment Model" or EPIS or "Dynamic Sustainability Framework" or "Practical, Robust Implementation and Sustainability Model" or "Promoting Action on Research Implementation in Health Services" or "Health Equity Implementation Framework" or "Evidence-Based Practice Attitude Scale" or "Program Sustainability Assessment Tool" or "Implementation Leadership Scale" or "Theory of Planned Behavio?r measures" or "Feelings Thermometer" or "Systems Usability Scale" or "ORgani#ational Social Context scale").tw,kf. |
| 5 | 1 or 2 |
| 6 | 3 or 4 |
| 7 | (checklist or scale* or self-report or survey* or questionnaire* or instrument* or tool* or measure* or "quantitative measure*" or "qualitative measure*" or interview* or "focus group*" or "open-ended questions" or "closed-ended questions" or observation or "discussion guide" or "participant observation" or "field notes" or log or diary or evaluation or "nominal group technique" or Delphi).tw,kf. |
| 8 | (adapt* or applicab* or suitab* or tailor* or modif* or translat* or appropriate* or validat* or "cross-cultural" or supplement* or compatab* or integrat* or alter* or adjust*).tw,kf. |
| 9 | 6 and 8 |
| 10 | 7 and 8 |
| 11 | 9 or 10 |
| 12 | exp "Minority Groups"/ or exp "racial groups"/ or exp ethnicity/ or exp "Ethnic Groups"/ or exp "indigenous peoples"/ or exp "health disparate minority and vulnerable populations"/ or "Ethnic and Racial Minorities"/ or "vulnerable populations"/ or "Transients and Migrants"/ or "emigrants and immigrants"/ or refugees/ or "Emigration and Immigration"/ or "health status disparities"/ or "healthcare disparities"/ |
| 13 | (underserved or underresourced or "under-resourced" or "underrepresented" or "under-represented" or "hard to reach" or "hard-to-reach" or minorit* or ethnic* or racial* or cultur* or ethnoracial or multiracial or linguistic* or ancestr* or decent or bicultural or multicultural or multi-cultural or "multi cultural" or intersectional* or CALD or "speaking background*" or NESB or bilingual* or non-English or non-European or non-white or non-Caucasian or "people of colo?r" or "persons of colo?r" or "communities of colo?r" or "global majority" or Black or "Afr* American*" or BIPOC or BAME or immigrant* or emigrant* or foreign* or refugee* or migrant* or asylum or "displaced people" or Indigenous or native* or "first nation*" or "first people*" or tribe* or tribal).tw,kf. |
| 14 | 12 or 14 |
| 15 | 5 and 11 and 14 |

Supplementary Material 3. Data extraction template for the FRAME and EVM variables

| **Variable** | **Instructions** | **Options** |
| --- | --- | --- |
| Was the adaptation proactive or reactive? (FRAME) | Select from the options | Proactive  Reactive |
| When did the modification occur in relation to data collection? (FRAME) | Select from the options | Before data collection  During data collection  After data collection |
| What was the goal for adapting the tool? (FRAME) | Select from the options and include justification | Increase reach or engagement  Increase retention Improve feasibility Improve fit with recipients To address cultural factors Improve effectiveness / outcome Reduce cost Increase satisfaction |
| What were the reason(s) for adapting the tool? (FRAME) | Copy and paste from the article |  |
| Who participated in the decision to adapt the tool? (FRAME) | Select from the options  Was a positionality statement included? Or how did the authors describe the decision makers | Political leaders Funder Administrator Research team Program manager Intervention developer Individual practitioners who deliver the intervention Community members |
| For whom (at what level) was the adaptation made? | Select from the options | Individual  Target intervention group  Cohort/individuals who share a particular characteristic  Individual researcher/practitioner  Service level  Network/system/community |
| What was the nature of the adaptation? (FRAME) | Select from the options  **and describe** | Tailoring/tweaking/refining Changes in packaging or materials Adding elements Shortening/condensing Lengthening/extending Substituting Reordering items Loosening structure |
| Describe the methods, processes used to inform the adaptation process | Describe and summarise | n/a |
| Who participated in the adaptation process? (FRAME) | Select from the options and describe, including number of participants, sampling strategy and recruitment | Political leaders Funder Administrator Research team Program manager Intervention developer Individual practitioners who deliver the intervention Community members |
| What was adapted? (EVM) | Select from the options and describe | **Language** e.g., translation **Persons** e.g., from provider to community **Metaphors** e.g., specific parts of the language related to the metaphors **Content** e.g., number of items, order **Concepts** e.g., specific parts of the language related to concepts **Goals** e.g., to guide more culturally appropriate implementation **Methods** e.g., survey to focus group **Context** e.g., adapting from hospital to community setting |


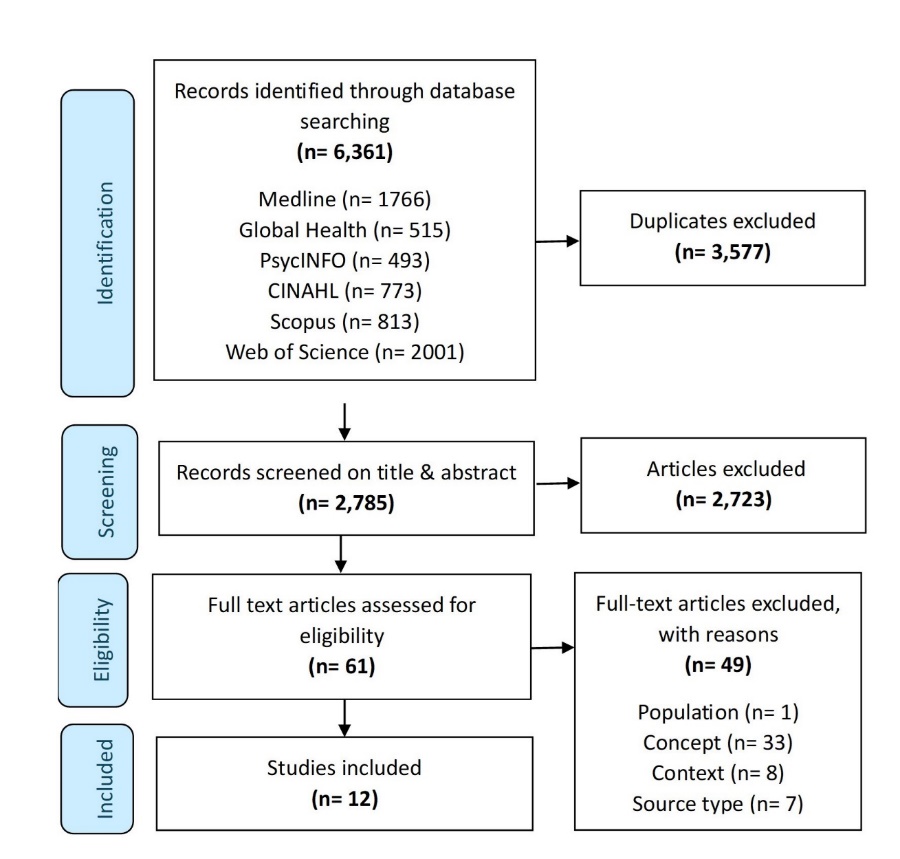
Supplementary Material 4. PRISMA diagram showing the flow of records from identification to inclusion in the scoping review.

Supplementary Material 5. Application of the studies, assessment of impact and further testing

| **Study**  **Year** | **Adapted tool** | **Applied** | **Participants** | **Methods** | **Impact assessed** | **Outcome(s) were used to evaluate the impact** | **Impact of the adaptation** | **Further testing** |
| --- | --- | --- | --- | --- | --- | --- | --- | --- |
| Barker 2025 | CFIR + First Nations Perceptions of Health and Wellness model | Yes | First Nations people of British Colombia who inject drugs (*n*=20)  Health planners (*n*=4) | Semi-structured CFIR + cultural model informed interviews   Coded using the nonadopted CFIR domains. Second cycle coding drew on the First Nations Perceptions of Health and Wellness model | No | n/a | n/a | No |
| Etherington  2020 | TDF + intersectionality lens | Yes | Nurses and other hospital staff e.g., nurses (*n*=NR) | Retrospectively applied as a case example to a barriers and enablers (interviews and survey) study about geriatric mobilisation and falls prevention perceptions at four hospital sites | No | n/a | Authors note: The tool draws attention to intersecting social factors and may encourage users to reflect on their implications on a wider scale.  Our tool can encourage users to think about interactions between domains (e.g. between “identity” factors and “social influences”) and how power structures may play a role in these interactions.  The prompts proposed in our enhanced version of the TDF may help to improve the quality of the information gained when conducting TDF surveys or interviews. | No |
| Gustafson 2024 | EquIR + cultural context = new model | No | n/a | n/a | No | n/a | n/a | No |
| Hikaka 2024 | RAPT + equity considerations for developing trials with Indigenous Peoples = RAPT-I | No | n/a | n/a | Yes | Participants involved in the adaptation process were asked if they would use the adapted tool | 72% responded yes  Authors note: Promoted culturally safe interventions and research practices and help researchers to identify areas to strengthen before a trial.  Provide a framework for critical review by funders and ethics board. | No |
| Jacobs  2023 | CFIR + Culturally Responsive Evaluation Framework | Yes | Program leaders, nutritionists, referral partners, clinical staff, community partners, lifestyle coaches and health educators (*n*=NR) | Evaluation of a diabetes prevention program including interviews and focus groups | No | n/a | Authors note: We considered the contextual and cultural factors included in the community (or outer setting) and within the organisations implementing the program (inner setting) characteristics of the program staff and priority populations | No |
| Johnson 2024 | CFIR-based focus groups + photovoice | Yes | Latino (*n*=12) and non-Latino - African American, Afro-Caribbean, American Indian/Alaskan, Multi-racial, White (*n*=12) men who have sex with men | Photovoice study with themes analysed using the CFIR | No | n/a | Authors note: Although the CFIR and implementation science methodology allowed us to identify implementation barriers and facilitators within specific domains, the findings also indicated that the CFIR may be limited in specifying the intersections among these domains that might reveal other culturally relevant factors influencing implementation. The CFIR may not adequately encompass cultural differences between researchers and participants. | No |
| Robak  2024 | TDF + cultural constructs | Yes | Community leaders, program administrators, community (*n*=9)   Physicians, nurses, allied health (*n*=11)  Community workshops (*n*=NR) | Semi-structured interviews using the modified TDF  Coded deductively to the TDF then inductively  Community workshop with community leaders and prospective service users to sense check | No | n/a | Authors note: Despite revising the TDF to enrich our understanding of Indigenous knowledge ‘Culture’ and ‘Environmental, Context & Resources’, our study found it inadequate in considering Indigenous cultural perspectives about healthcare delivery. This inadequacy may stem from reductionist notions of health prevalent in biomedicine compared to Indigenous models of health. Our modifications may serve as foundations for future revisions of the TDF to capture important contextual enablers and barriers when implementing initiatives in similar settings. While the TDF provided a viable scaffold, further cultural adaptation is needed to appropriately assess local health systems and care practices in Indigenous communities in Canada. | No |
| Rodrigues 2023 | CFIR + Intersectionality lens | No | n/a | n/a | No | n/a | n/a | No |
| Sebastian 2021 | CFIR + cultural suitability construct | Yes | Healthcare professionals, Indigenous (*n*=12), non-Indigenous (*n*=12) | Semi-structured interviews | No | n/a | Authors note: The newly introduced construct allowed for deeper probing into the versatility of the different training components in ensuring their suitability for the Indigenous trainee and client population. | No |
| Senier  2019 | CFIR + fundamental cause theory | Yes | Multi-ethnic communities including Latinx and Chinese | Use case examples of how the proposed framework is applicable | No | n/a | Authors note: We show that the CFIR and fundamental cause theory frameworks are highly compatible and can encourage implementation efforts that are sensitive and responsive to threats of health inequities, whether they arise from intra-clinic or external factors. | No |
| Thompson2022 | EPIS + trans health context = new model | No | n/a | n/a | No | n/a | n/a | No |
| Woodward 2019 | i-PARIHS + the Health Care Disparities Framework = Health Equity Implementation Framework | Yes | Black, Southern, rural-dwelling, older adult male Veterans Affair patients diagnosed with Hepatitis C (*n*=12) | Semi-structured interviews  Directed content analysis based on the framework for top level codes. Then data mined for concepts about racial disparities (experiences, perceptions, or reasons for differences in treatment between Black and white patients) | Yes | Feasibility of the framework for researchers | Feasibility - not defined or measured in a systematic way. Authors note: Requires researcher knowledge and time to generate the interview guide.  Authors note: Allowed for systematic assessment of barriers and facilitators and sources of inequities specific to the group while simultaneously capturing barriers that were either independent of, or co-occurring with, typical implementation barriers. Requires community engagement is required to have the knowledge of what to assess for each domain. | No |

Abbreviations: n/a, not applicable (if an answer is No, the subsequent boxes are marked n/a); CFIR, Consolidated Framework for Implementation Research; TDF, Theoretical Domain Framework; RAPT-I Model, Readiness Assessment for Pragmatic Trials Model -Indigenous, TMFs, Theories, Models, Frameworks; EPIS Framework, Exploratory, Preparation, Implementation, Sustainment
